# Brachial Plexus Block for Removal of Retained Radial Artery Sheath

**DOI:** 10.7759/cureus.33068

**Published:** 2022-12-28

**Authors:** Peter L Kovacs, Zachary Deutch, Daniel Castillo

**Affiliations:** 1 Department of Anesthesiology, University of Florida College of Medicine, Jacksonville, USA

**Keywords:** ultrasound-guided regional anesthesia, coronary artery angiography, upper extremity sympathectomy, retained radial artery sheath, supraclavicular brachial plexus block

## Abstract

In this case, we present a lesser-known application of regional anesthesia, specifically, managing a patient with vasospasm and retained radial artery (RA) vascular sheath after coronary angiography. Providing an ultrasound-guided supraclavicular block, in combination with general anesthesia, allowed the proceduralist to remove the retained sheath after several hours of failed treatment and manipulation. Severe arterial spasm was alleviated by eliciting a sympathectomy, along with analgesia of the right upper extremity, and maintaining this post-procedure. The block optimized arterial flow through the RA post-intervention and helped manage the patients’ pain from manipulation.

## Introduction

Radial artery (RA) spasm is a known side effect of manipulation of the vessel, for example during catheterization and harvesting. As a result, systemic and local vasodilators (including calcium channel blockers, nitroglycerine, and papaverine) are administered [1].

Upper extremity sympathectomy from brachial plexus block has been shown to reduce vasospasm of the RA, and the block's utility in other vascular procedures (e.g. creation of arterio-venous fistulas) has also been noted. A pre-emptive sympathectomy prevents vasospasm intraoperatively, and this protective effect continues into the immediate postoperative period [2-5]. We present a case of severe RA vasospasm after the percutaneous cardiac intervention, causing undesired retention of the intra-arterial sheath. The sheath was removed under a supraclavicular, ultrasound-guided brachial plexus block and general anesthesia (GA).

## Case presentation

A 78-year-old Caucasian female with a past medical history of diabetes mellitus, hyperlipidemia, hypertension, prior cerebral vascular accident, hypothyroidism, and coronary artery disease presented to the emergency department complaining of a “funny feeling in her head,” visual changes, and lightheadedness. She had no focal neurological deficits and denied any loss of consciousness. She was compliant with a medication regimen consisting of aspirin, proton pump inhibitor, statin, hydrochlorothiazide, thyroid hormone replacement, beta-blocker, and angiotensin-converting enzyme (ACE) inhibitor. Vital signs were within normal limits, she was in no acute distress, and the physical exam was without remarkable findings. Initial lab work revealed normal cardiac enzymes, hemoglobin level, and white cell count. A cardiac work-up two months prior revealed severe left circumflex disease, an ejection fraction of 75%, and a small area of possible ischemia in the mid and anterolateral ventricular wall. The patient was diagnosed with angina unresponsive to medical treatment and was scheduled for coronary angiography.

The patient was medicated with 1 mg of midazolam and 1 mg of hydromorphone intravenously. The right RA was selected for access following a modified Allen's test and doppler ultrasound interrogation. A 6 French Hydrorophilic Transradial sheath 0.018 (Vascular Solutions, Minneapolis, MN) was inserted without difficulty. Through the sheath, 3 mg of verapamil and 3000 units of heparin were given. Because of difficulty directing a 5 Fr JR 3.5 catheter to the right coronary artery, along with patient complaints of pain on catheter manipulation, the short sheath was removed over a wire. A 6 Fr x 65 cm destination sheath was then inserted to facilitate procedural manipulation and reduce irritation. An additional 3 mg of verapamil was given intra-arterial before re-insertion of the sheath.

The patient continued to complain of brachial and forearm pain and was treated with topical nitroglycerin to the forearm area, treating presumptive spasms. Additional midazolam and hydromorphone were also given.

**Figure 1:**
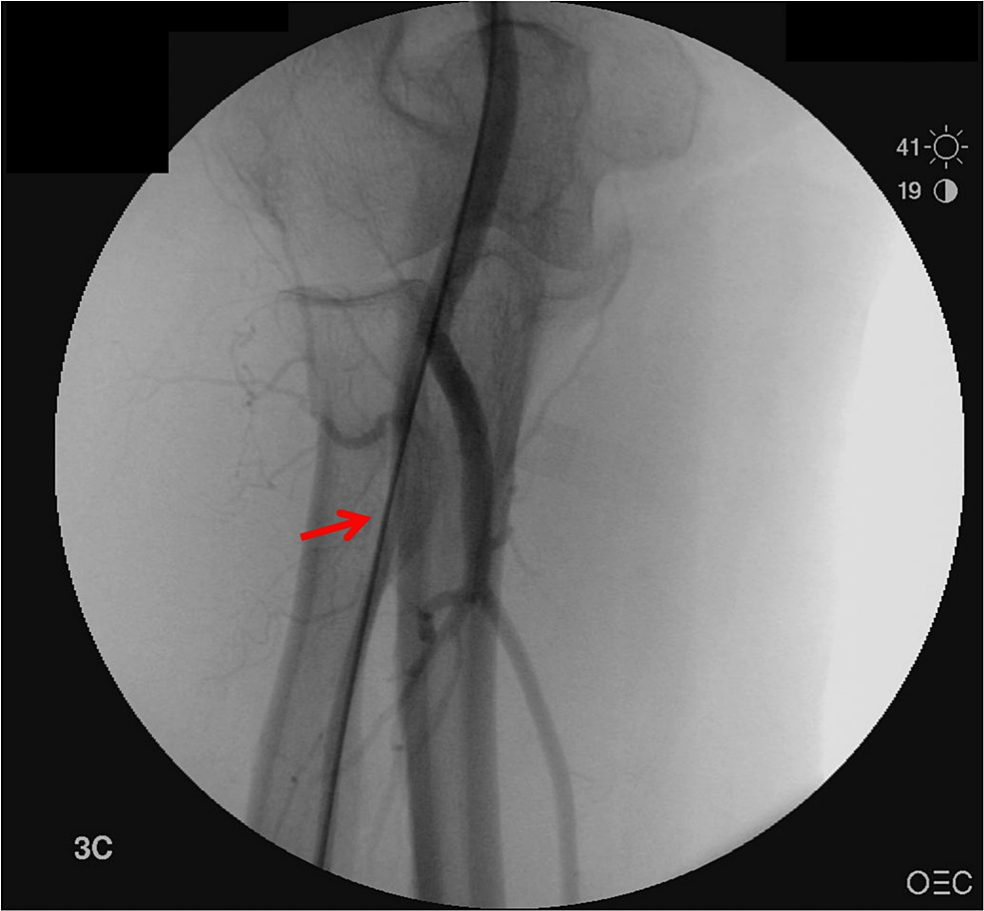
Angiography of right upper extremity showing spasm in radial artery containing the vascular sheath (red arrow)

On completion of the procedure, there was significant resistance when trying to remove the long radial sheath; meanwhile, the patient complained of excruciating pain as this was attempted (Figure 1).

Lidocaine, verapamil, and nitroprusside were unsuccessfully administered through the sheath in an attempt to induce local vasodilation. The patient was transferred to the CCU (coronary care unit) on heparin infusion, topical nitroglycerin to the brachial area, and intravenous analgesia, with the intent of awaiting the resolution of the vasospasm. Warm compresses were applied, and no further systemic vasodilators were given since the procedural team was concerned about the patient becoming hypotensive. No features of distal cyanosis were visible.

**Figure 2:**
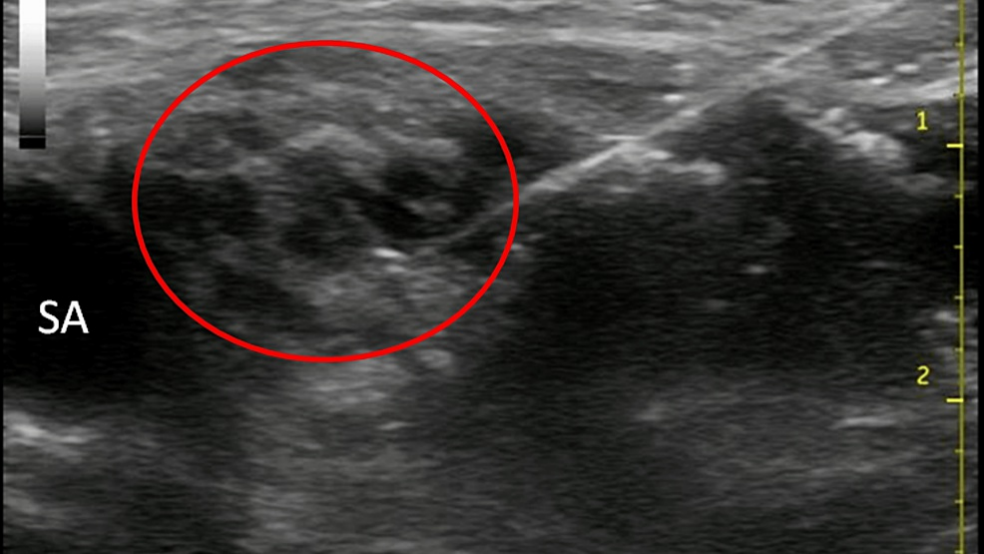
Image of supraclavicular nerve block showing the subclavian artery (SA), brachial plexus (circled), and the block needle

After four hours, removal of the radial sheath was still not possible with gentle traction in the CCU and the anesthesiology department was consulted to assist in management. Our recommendation was to attempt the removal of the sheath under the brachial plexus block. The patient was brought to the pre-op holding area and a supraclavicular nerve block was placed. The skin of the right supraclavicular area was prepped with betadine and draped with sterile towels. Using ultrasound guidance with a General Electric Logic-e portable ultrasound machine with a 12L linear array probe (GE Healthcare, Wauwatosa, WI) the subclavian artery and the brachial plexus were identified, as well as the pleura and the first rib. A two-inch 22g block needle (Braun Stimuplex, Bethlehem, PA) was advanced under direct visualization to the vicinity of the brachial plexus, and, after negative aspiration, 20cc of 0.2% ropivacaine was injected slowly, obtaining good spread around the nerves (Figure 2).

**Figure 3:**
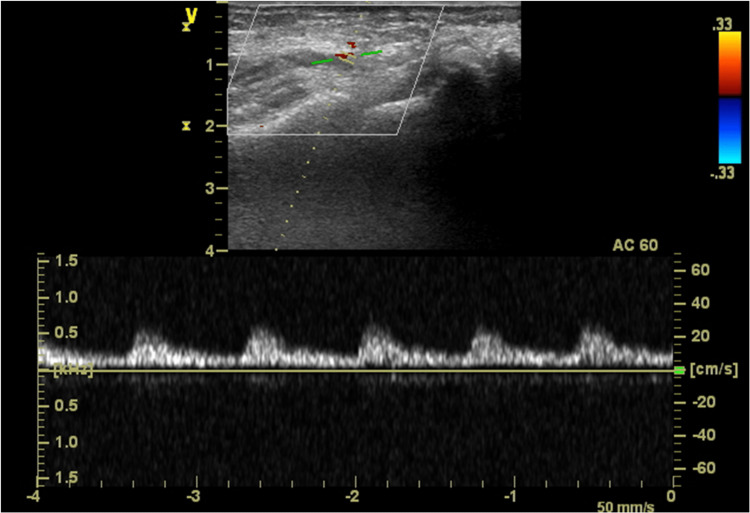
Right radial artery flow with ultrasonography post spasm and removal of the vascular sheath

The patient was then taken to the operating room, while simultaneously she stated that her pain was resolving. Proceeding with attempted removal under mild sedation was considered, but, at the cardiology team’s request, GA was induced, due to uncertainty about how the intra-operative course would play out. The patient was induced using intravenous medications (lidocaine 50 mg, propofol 150 mg, and midazolam 2 mg) and a #4 laryngeal mask airway was easily placed. Sevoflurane was used to maintain anesthesia, and no narcotics were given. Angiography was again performed, now demonstrating resolution of the spasmodic segment, with excellent flow through the artery. The long sheath was removed with minimal resistance and the RA integrity was maintained with no retaining products. The patient was taken to the recovery room stable and in no pain, with a good palpable right radial pulse (Figure 3).

## Discussion

The RA is a spastic, highly reactive vessel due to its physiological, anatomical, and embryological characteristics. It is a type 3 vessel based on He’s classification [6]. Its pharmacologically induced contractility is more pronounced than other arterial conduits. Mechanical manipulation readily induces spasms, which can lead to A-V fistula construction failure as well as early graft failure after coronary artery bypass graft (CABG) [7]. The RA is a “temperamental” vessel, which, as a result of this nature, has been debated as a proper conduit in CABG surgery. Though all arterial conduits display equal endothelial function, the RA has more pronounced angiotensin II and endothelin 1 induced contractility than the internal mammary artery (IMA) [8]. The RA is an alpha-adrenoreceptor dominant artery (mostly Alpha 1 subtype) and is therefore extraordinarily sensitive to phenylephrine infusion during cardiac surgery [9,10].

Several interventions have been suggested to reduce the RA's tendency to spasm, namely nitroglycerin, calcium channel blockers, and phentolamine (oral or intravenous), but their use is controversial, and their efficacy has been equivocal [11-13]. In this particular case, it was markedly difficult to retrieve the long catheter from the RA because of arterial spasm and pain, which underscores the proclivity of and magnitude with which this artery can contract. In this case, nitroglycerin and phentolamine were given both systemically and locally, yet were ineffective.

An ultrasound-guided supraclavicular block was eventually performed, with the goal of creating an upper extremity sympathectomy [14]. GA was also induced, though we felt that the catheter could have been removed under regional anesthesia alone. Because this had been a significant, concerning problem for a period of hours, the cardiology team did not want to wait for the block to be fully set up and asked us to provide GA urgently. The team was also unsure what would happen to the patient on the second attempt at removal. Regardless, it is well known that GA produces vasodilatation, which was likely beneficial in this case. Having a brachial plexus block in place alleviated concerns about hand ischemia due to post-surgical manipulation or post-emergence spasm since the vasodilatory effects of GA disappear on emergence, but the regional sympathectomy due to upper extremity nerve block lasts for a period of hours. Ultimately, the catheter was easily removed, and continuous blood flow to the hand was present after emergence from GA, with a normal capillary refill, easily palpable pulses, and normal hand color.

Although retention of a radial sheath is uncommon, it has been described after angiographic procedures [15,16]. Complications from retained RA catheters have been reported to include persistent bleeding, cannulation site infection, and granuloma [17-19]. A technique to consider when putting large bore, and/or lengthy catheters into the RA, with the possibility of multiple exchanges or cannulation attempts, is to perform regional anesthesia, either alone, or in combination with GA, in order to ensure continuous vasodilation either during or after the procedure. Peripheral nerve blocks causing bleeding complications such as hematoma formation are rare in patients receiving antiplatelet and/or anticoagulant medication [20]. Regional anesthesia should also be considered as a rescue technique for a retained catheter due to vasospasm.

## Conclusions

We present a case and subsequent treatment of a retained vascular sheath in the RA of a patient undergoing cardiac catheterization to assess coronary artery function. A technique to consider when placing large bore, and/or lengthy catheters into the RA, with the possibility of multiple exchanges or cannulation attempts, is to perform regional anesthesia, either alone or in combination with GA, in order to ensure continuous vasodilation during and after the procedure.
